# Fatal Case of Enterovirus 71 Infection, France, 2007

**DOI:** 10.3201/eid1511.090493

**Published:** 2009-11

**Authors:** Sophie Vallet, Marie-Christine Legrand-Quillien, Thomas Dailland, Gaëtan Podeur, Stéphanie Gouriou, Isabelle Schuffenecker, Christopher Payan, Pascale Marcorelles

**Affiliations:** Université Européenne de Bretagne, Brest, France (S. Vallet, G. Podeur, S. Gouriou, C. Payan); Centre Hospitalier Universitaire, Brest (S. Vallet, M.-C. Legrand-Quillen, T. Dailland, C. Payan, P. Marcorelles); Centre de Biologie et Pathologie Est, Bron, France (I. Schuffenecker)

**Keywords:** Enterovirus 71, pulmonary edema, encephalitis, viruses, France, dispatch

## Abstract

A fatal case of enterovirus 71 infection with pulmonary edema and rhombencephalitis occurred in Brest, France, in April 2007. The virus was identified as subgenogroup C2. This highly neurotropic enterovirus merits specific surveillance outside the Asia-Pacific region.

Enterovirus 71 (EV71), like other enteroviruses (family *Picornaviridae*), induces mostly asymptomatic or clinically benign infections. The virus is a leading cause of foot and mouth disease and, above all, is an emerging agent of acute central nervous system disease (aseptic meningitis, flaccid paralysis, encephalitis). Since its identification in 1969, EV71 has been the subject of several studies, particularly over the past decade in the Asia-Pacific region where several outbreaks have been reported (*1*–*5*). Epidemics have also been described in the United States, Brazil, and Europe (Sweden in 1974, Bulgaria in 1975, and Hungary in 1978) (*6*). EV71 can be divided into 3 independent genogroups by molecular typing: A, B, and C (*1*). Genogroups B and C can be further subdivided into subgenogroups B1–B5 and C1–C5 (*6*). Subgenogroups can replace each other within a short period of time, but several genotypes can also cocirculate as seen in Malaysia (*2*).

Death caused by this virus occurs rarely; young children are especially at risk. This report describes a fatal case of acute pulmonary edema with rhombencephalitis that occurred within the course of an EV71 infection in France.

## The Case-Patient

In April 2007, a 17-month-old boy was referred to the pediatric emergency ward of the Brest University Hospital. He had hyperthermia, which had begun 48 hours earlier. Upon arrival, he appeared to be in good general condition, despite mild respiratory discomfort and episodes of vomiting. Nasopharyngitis was diagnosed. He was discharged with treatment consisting of symptomatic medication and oral rehydration.

He was readmitted to the pediatric emergency unit 12 hours later, in severe respiratory distress. Disorders of consciousness and drowsiness were observed. He was immediately given supportive intravenous corticotherapy in association with aerosol and oxygen therapy. He was subsequently transferred to the pediatric intensive care unit. Results of his chest radiograph were normal. Laboratory test results were as follows: leukocytosis 23,300/mm^3^ (67.5% polynuclear neutrophils), platelets 453,000/mm^3^, hemoglobin 116 g/L, and hematocrit 34.9%. The C-reactive protein value was elevated (11 mg/L) and liver function was conserved. Severe dyspnea, hyperglycemia (2.02 g/L), and a decreased blood pressure (38 mm Hg) rapidly developed. After the onset of this acute respiratory distress syndrome, a second chest radiograph showed marked lung infiltration. The patient was intubated because of increased oxygen dependence. Cardiorespiratory arrest occurred during the induction of anesthesia. Despite cardiopulmonary resuscitation, he died <12 hours after his second admission to the pediatric emergency ward.

**Figure 1:**
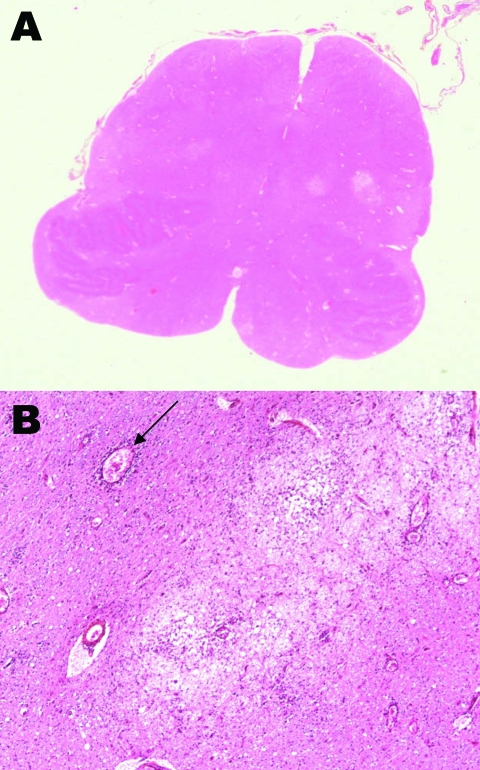
A) Horizontal section of the medulla at the level of the inferior olivari nuclei, showing multiple inflammatory areas (clear areas); original magnification ×4. B) Severe edematous area with inflammatory cells, macrophages, edema, and perivascular cuffing (arrow); original magnification x200, hematoxylin-eosin stain.

An autopsy was performed with the prior consent of the parents. Pulmonary edema and multiple foci of polymorph inflammatory infiltrate were present in lung samples. Encephalitic necrotic lesions were multifocal but predominant in the inferior brainstem and superior cervical medulla. Respiratory centers were affected, as were vegetative nucleates of the medulla oblongata (Figure 1). No inflammatory or necrotic areas were found in cardiac muscle.

**Figure 2:**
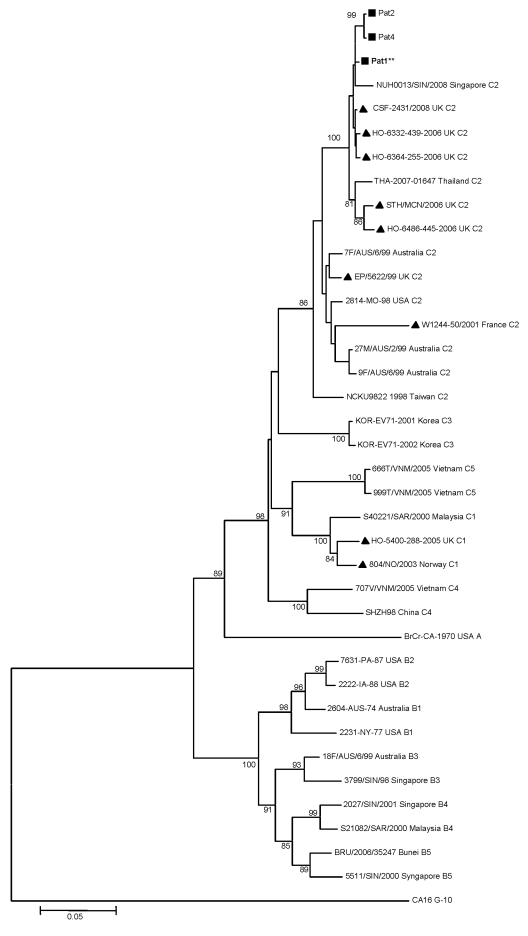
Phylogenetic relationships between 3 French strains and 34 worldwide enterovirus 71 GenBank-selected strains based on alignment of complete viral protein (VP) 1 coding sequences. The prototype coxsackievirus A16 (CoxA16-G10) was used as the outgroup virus. The phylogenetic tree was constructed by the neighbor-joining method by using MEGA4 (www.megasoftware.net). Bootstrap values (>70%) derived from 1,000 samplings are shown at the nodes of the tree. Phylogenetic separation of C2 isolates appear in accordance with time and place of isolation. Isolates from this study are indicated by black squares (the strain isolated from the 17-month-old boy with fatal pulmonary edema is shown in boldface with **), and the other European circulating strains by black triangles. Branch lengths are drawn to scale. Scale bar indicates 5% of nucleotide sequence divergence.

An enterovirus was isolated in the MRC-5 cell culture from a bronchial aspirate taken just before death. An enterovirus was also isolated in an autopsy nasal swab. Other autopsy samples (lung, spleen, and urine) were positive for enterovirus by reverse transcription–PCR (RT-PCR) (Enterovirus consensus kit, Argene, Verniolle, France), but no virus was isolated from these samples. Brain tissue could not be used for virologic studies because of formalin fixation. The enterovirus strain isolated in cell culture was sent to the French National Reference Centre for Enteroviruses, where it was identified as EV71 by partial viral protein (VP) 1 sequencing (*7*). A second analysis was performed that targeted the 891 nucleotides of the VP1 gene, as described by Bible et al. (*8*). This sequence was compared by phylogenetic analysis with 34 GenBank-selected VP1 enterovirus 71 strains (Table). A phylogenetic tree was constructed by neighbor-joining, using the Kimura 2-parameter distance method to define relationships between the current isolate and other EV71 subgenogroups. The VP1 sequence of the French patient who died clustered with VP1 sequences belonging to the C2 subgenogroup (Figure 2). The 2 closest-matching isolates originated from 2 young children admitted to the Brest Hospital emergency ward a few months later, in June and July 2008, respectively. The first, 13 months of age, was admitted for acute respiratory syndrome, which rapidly resolved. Enterovirus 71 was isolated from the child’s bronchial secretions. The second child, 31 months of age, had acute gingivostomatitis, fever, and cerebellar syndrome evocative of meningoencephalitis. Enterovirus was isolated from buccal lesions, but no virus was detected by RT-PCR in cerebrospinal fluid (CSF), even though pleocytosis was present. He recovered quickly. The VP1 full-length sequences of the 3 strains were deposited in GenBank under the accession nos. FJ824734–FJ824736. The high nucleotide identity among the 3 strains (>98%), and their place and period of circulation suggest a probable identical ancestral strain, which remains undefined. The origin of the EV71 strain responsible for the substantial pulmonary edema described here remains unknown. The 3 strains isolated clustered with 3 other European strains, isolated in 2006 and 2008 in the United Kingdom (*8*,*9*), and with 2 Asian strains, isolated in 2007 in Thailand and in 2008 in Singapore and Thailand (GenBank, unpub. data).

**Table:** EV71 clinical strains used in phylogenetic analysis of the complete VP1 gene*

GenBank accession no.	Year	Origin	Clinical features	Isolate	Genogroup	Reference
U22521	1970	USA	Encephalitis	BrCr-CA-70	A	(*1*)
AF135883	1974	Australia	Meningitis	2604-AUS-74	B1	(*1*)
AF135870	1977	USA	NA	2231-NY-77	B1	(*1*)
AF009533	1987	USA	Gastroenteritis	7631-PA-87	B2	(*1*)
AF009540	1988	USA	Fever	2222-IA-88	B2	(*1*)
AF376117	1998	Singapore	HFMD	3799/SIN/98	B3	(*2*)
AF376095	1999	Australia	HFMD	18F/AUS/6/99	B3	(*2*)
AF376084	2000	Sarawak	HFMD	S21082/SAR/2000	B4	(*2*)
AF376111	2000	Singapore	NA	2027/SIN/2001	B4	(*2*)
AF376121	2000	Singapore	HFMD	5511/SIN/2000	B5	(*2*)
FM201341	2006	Brunei	NA	BRU/2006/35247	B5	(*5*)
AF376087	2000	Sarawak	HFMD	S40221/SAR/2000	C1	(*2*)
DQ452074	2003	Norway	NA	804/NO/2003	C1	(*10*)
AM939598	2005	UK	Unspecified neurologic disease	HO-5400-288-05	C1	(*8*)
AF135950	1998	Missouri	Meningitis	2814-MO-98	C2	(*1*)
AF136379	1998	Taiwan	Death	NCKU9822	C2	(*3*)
AM939583	1999	UK	Aseptic meningitis	EP/5622/99	C2	(*8*)
AF376108	1999	Australia	Meningitis	7F/AUS/6/99	C2	(*2*)
AF376102	1999	Australia	HFMD	27M/AUS/2/99	C2	(*2*)
AF376110	1999	Australia	Ataxia	9F/AUS/6/99	C2	(*2*)
AY208099	2001	France	Guillain-Barré syndrome	W1244-50/2001	C2	(*11*)
AM939604	2006	UK	Meningitis	HO-6332-439-06	C2	(*8*)
AM939607	2006	UK	Irritable, rash	HO-6364-255-06	C2	(*8*)
AM939597	2006	UK	Panencephalitis, fatal	STH/MCN/06	C2	(*8*)
AM939599	2006	UK	Difficulty walking	HO-6486-445-06	C2	(*8*)
FJ461786	2008	Singapore	NA	NUH0013/SIN/2008	C2	UD
FJ151492	2007	Thailand	NA	THA-07-01647	C2	UD
FJ525952	2008	Edinburgh	NA	CSF-2431/2008	C2	(*9*)
AY125966	2001	Korea	NA	KOR-EV71-01	C3	UD
AY125967	2002	Korea	NA	KOR-EV71-02	C3	UD
AF302996	1998	China	NA	SHZH98	C4	UD
AM490154	2005	Vietnam	NA	707V/VNM/2005	C4	(*4*)
AM490163	2005	Vietnam	NA	999T/VNM/2005	C5	(*4*)
AM490153	2005	Vietnam	NA	666T/VNM/2005	C5	(*4*)

*EV, enterovirus; VP, viral protein; HFMD, hand, foot, and mouth disease; NA, not available; UD, unpublished data.

## Conclusions

Acute pulmonary edema in EV71 infections was rarely reported before the 1998 outbreak in Taiwan (*12*). Since then, this disease, which is often fatal, has been more frequently described, with known prognostic factors, including clinical and biologic features such as central nervous system involvement, leukocytosis, decreased blood pressure, and hyperglycemia (*13*). The fatal infection reported here, which ran a biphasic course, featured all of these signs, with death occurring within a few hours of the onset of respiratory distress. This devastating syndrome is believed to result from the extensive damage of bulbar vasomotor and respiratory centers (Figure 1). In the study by Kao et al. all 21 patients who had acute pulmonary edema associated with these signs died within 4 hours of the development of acute respiratory distress syndrome (*13*).

No reports of fatal cases of EV71 infection in Europe have been made since cases were reported in Hungary in 1978 (*9**–**11**,**14*) with the exception of a fatal case of panencephalitis associated with subgenogroup C2 in the United Kingdom (*8*). No single neurovirulent genotype appears to be associated with severe and fatal cases; at least 3 separate genotypes have been isolated from patients with fatal cases in Malaysia (Sarawak), Japan, and Taiwan (*6*). A similar subgenogroup such as C1 may be associated with complicated disease in Sarawak and asymptomatic infection in Norway (*2*,*10*). A specific marker for virulence has yet to be defined.

Further studies are required to estimate the prevalence of EV71 infection, and only extensive clinical, virologic, and anatomopathologic investigation may determine the actual prevalence of EV71 in severe and sometimes fatal neurologic diseases. EV71, like poliovirus, is not always recovered from CSF during central nervous system infection (*15*). This fact underscores the importance of virologic investigations on peripheral samples (throat swabs, urine, stool, and vesicles).

Since 2006, centers participating in the French Enterovirus Surveillance Network have declared a significantly growing number of EV71 infections (D. Antona, pers. comm.). The international spread of EV71 outside the Asia-Pacific region needs to be vigilantly monitored, because both the possibility of an outbreak and the unpredictability of virus circulation patterns represent a public health concern.
