# Erratum—Vol. 15, No. 11

**DOI:** 10.3201/eid1512.999000

**Published:** 2009-12

A word was missing from a sentence in the article Fatal Case of Enterovirus 71 Infection, France, 2007 (Vallet S, et al.). The second sentence of the article text should read: “The virus is a leading cause of hand, foot, and mouth disease and, above all, is an emerging agent of acute central nervous system disease (aseptic meningitis, flaccid paralysis, encephalitis).” The article has been corrected online (www.cdc.gov/eid/content/15/11/1837.htm).

